# Concurrent Neural and Vascular Variations in the Gluteal Region Involving the Sciatic Nerve, Inferior Gluteal Nerve, and Gluteal Arteries: A Cadaveric Case Report

**DOI:** 10.7759/cureus.98461

**Published:** 2025-12-04

**Authors:** Olivia G Hubbard, Joshua L Miles, Dillon T Duplechan, Ryson J Shelton, Hosne Ara, Adegbenro O Fakoya

**Affiliations:** 1 Anatomy, Louisiana State University Health Sciences Center, Shreveport, USA; 2 Cellular Biology and Anatomy, Louisiana State University Health Sciences Center, Shreveport, USA

**Keywords:** anomaly, inferior gluteal artery, inferior gluteal nerve, sciatic nerve, superior gluteal artery, surgery, variation

## Abstract

Anatomical variations in the gluteal region are not uncommon, but concurrent neurovascular anomalies are rarely reported. During routine cadaveric dissection, we identified a rare configuration in a 91-year-old male cadaver involving both neural and vascular variants. The sciatic nerve divides at the level of the piriformis, with the common fibular division piercing the muscle and the tibial division exiting below. Additionally, a branch of the inferior gluteal nerve also passed through the piriformis, accompanied by an anastomosis between the superior and inferior gluteal arteries. While each variation has been individually described in the literature, their simultaneous occurrence in a single gluteal region is uncommon. This case highlights the complexity of gluteal anatomy and underscores the importance of recognizing such variations during surgical planning, diagnostic imaging, and anatomical education to reduce iatrogenic risk and enhance clinical outcomes.

## Introduction

The gluteal region is a complex anatomical area that contains numerous clinically significant neurovascular structures, including the sciatic nerve, inferior gluteal nerve, and inferior gluteal artery [1,2]. The sciatic nerve is the largest in the human body, and it is critical for the motor and sensory innervation of the lower limb [1]. The inferior gluteal nerve innervates the gluteus maximus muscle and plays an essential role in hip extension, adduction, and lateral rotation [2]. Anatomically, the piriformis muscle serves as a key landmark for identifying adjacent structures in the gluteal region [3].

Typically, the sciatic nerve exits the pelvis through the greater sciatic foramen, emerging between the piriformis and superior gemellus muscles, and travels down the posterior thigh before dividing into the tibial and common fibular (peroneal) nerves near the popliteal fossa [1]. However, variations in the course and division of the sciatic nerve have been well documented [4]. One study compiled anatomical data from cadaveric literature and found that variants in the relationship between the sciatic nerve and piriformis muscle occurred in up to 17% of specimens [4]. The specific variation observed in our case, where the common fibular division pierces the piriformis while the tibial division exits below, was present in 13.6% of cadavers in their review [4]. In addition to this meta-analysis, the same study conducted magnetic resonance neurography (MRN) imaging and found this variation in 11.9% of scanned subjects [4].

Similar variations have also been reported for the inferior gluteal nerve, which normally exits the pelvis alongside the inferior gluteal artery below the piriformis [2]. Variants include the nerve being absent or taking an atypical course through or above the piriformis muscle [5]. The most common of these, in which the inferior gluteal nerve passes through the piriformis, has been observed in 15.2% of cadavers [5]. 

While isolated variations in either the sciatic or inferior gluteal nerves are not uncommon, concurrent variations involving both nerves in the same individual are rarely reported in the literature [4,5]. Al Talalwah described the sciatic nerve variation in detail, proposing a new classification to assist clinicians in differential diagnosis, and also documented the vascular variability of the superior and inferior gluteal arteries in the hip joint region [6,7]. This case report describes such a dual variation identified during routine cadaveric dissection, highlighting the anatomical complexity of the gluteal region and the importance of recognizing these anomalies in both clinical and educational settings.

## Case presentation

During a routine cadaveric dissection at Louisiana State University Health Sciences Center in Shreveport, Louisiana, medical students identified an unusual anatomical variation in the left gluteal region of a 91-year-old Caucasian male cadaver.

Dissection of the gluteal region began with the removal of the skin and superficial fascia. The gluteus maximus muscle was then reflected to expose deeper structures, followed by careful isolation and reflection of the gluteus medius muscle to avoid damage to the underlying neurovasculature. This revealed the piriformis muscle and its associated nerves and vessels.

**Figure 1:**
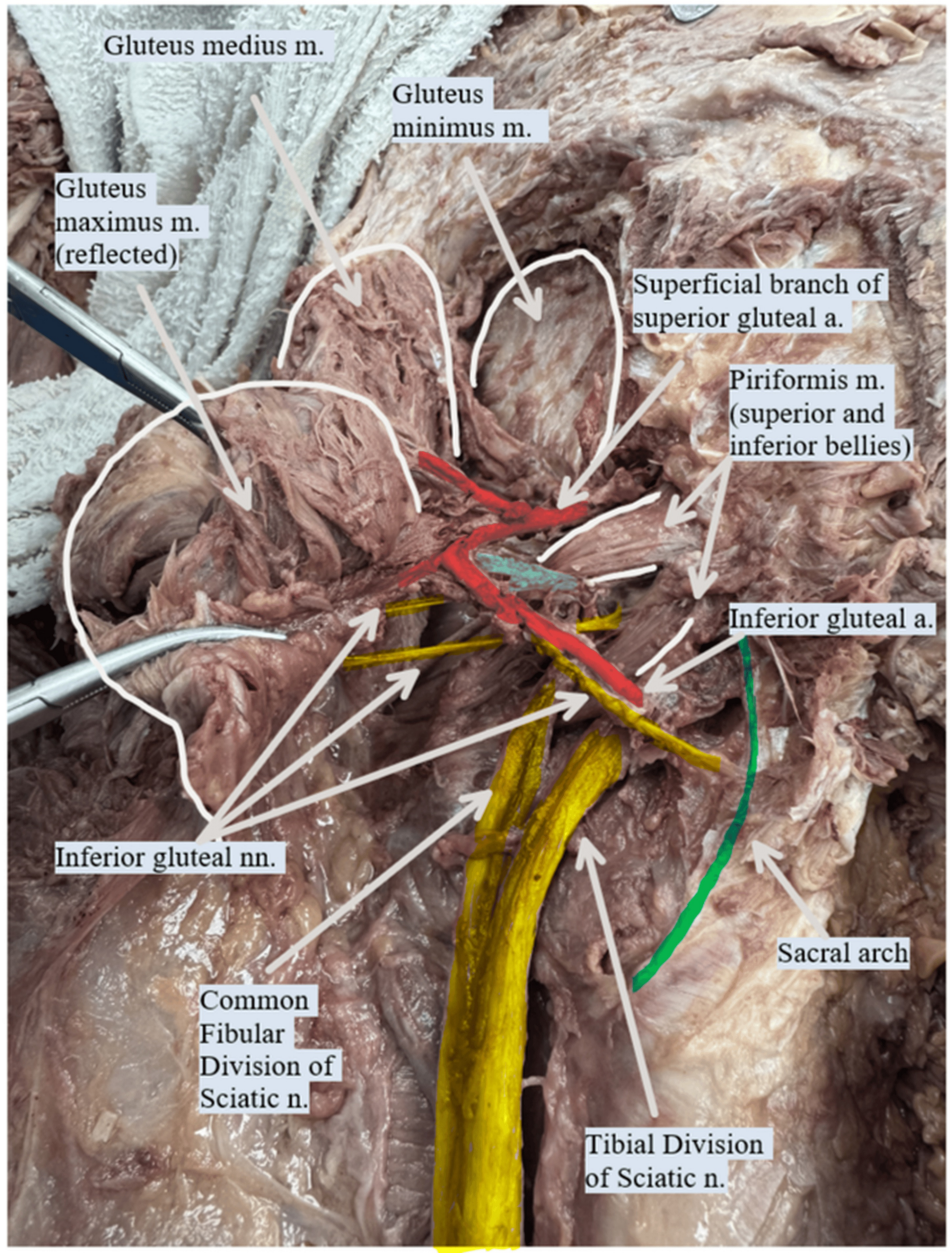
Anterior inferior view of the left gluteal region. This image shows that the piriformis muscle is divided into superior and inferior bellies. The common fibular nerve (yellow) emerges between the two bellies, while the tibial nerve (yellow) passes below the inferior belly. The branches of the inferior gluteal nerve (yellow) exit between the bellies and below the inferior belly. The inferior gluteal artery (red) exits below the inferior belly of the piriformis muscle to anastomose with the superficial branch (red) of the superior gluteal artery. m. - muscle; a. - artery; n. - nerve; nn. - nerves

**Figure 2:**
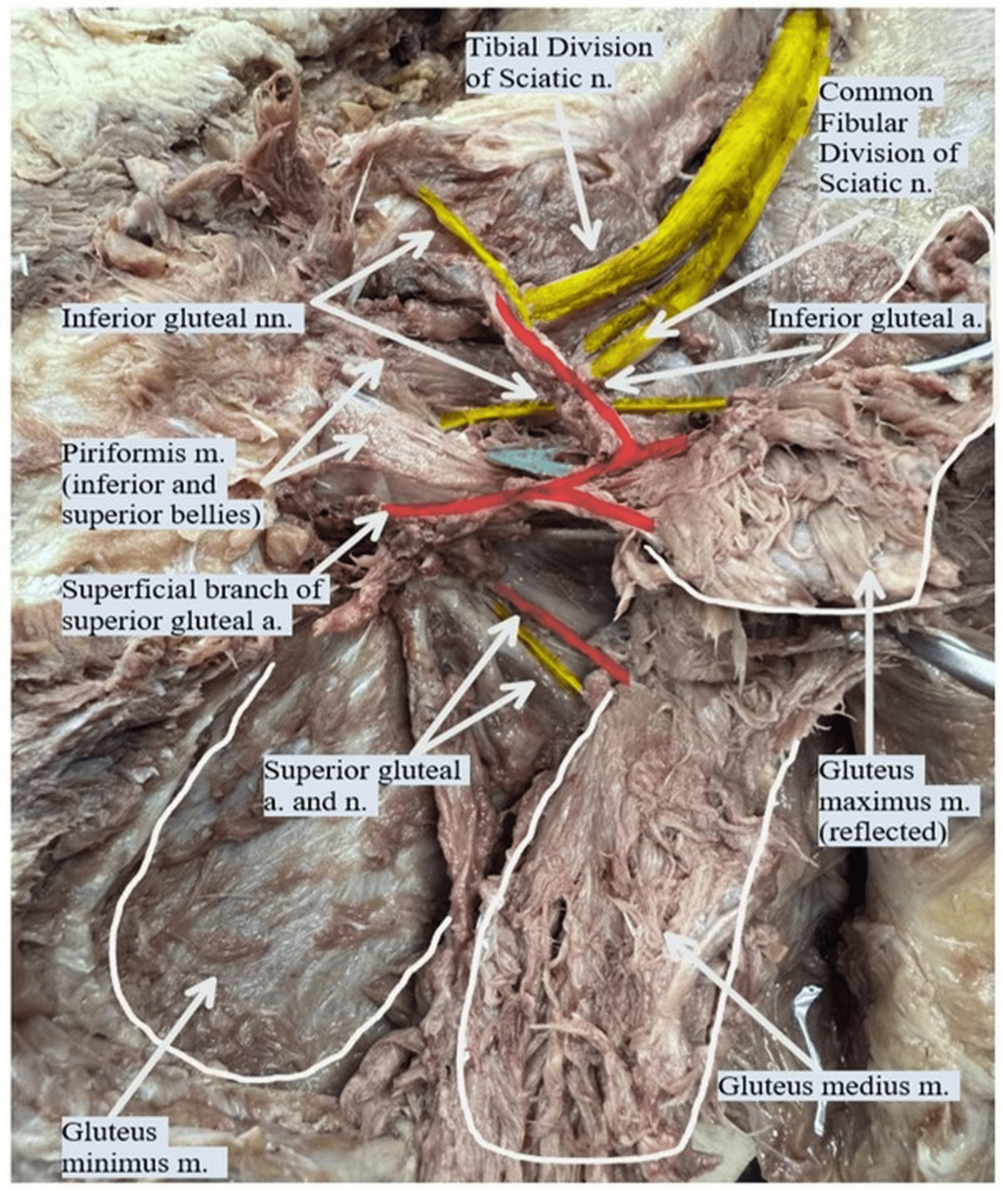
Bird's eye anterior superior view of the left gluteal region. This image shows the piriformis muscle divided into superior and inferior bellies. The common fibular nerve (yellow) emerges between the two bellies, while the tibial nerve (yellow) passes below the inferior belly. The branches of the inferior gluteal nerve (yellow) exit between the bellies and below the inferior belly. The inferior gluteal artery (red) exits below the inferior belly of the piriformis muscle to anastomose with the superficial branch (red) of the superior gluteal artery. The superior gluteal artery (red) exits above the superior belly of the piriformis to supply the gluteus medius and minimus. m. - muscle; a. - artery; n. - nerve; nn. - nerves

On examination of the left gluteal neurovascular anatomy, a distinct variation in the sciatic nerve was noted. The nerve was divided at the level of the piriformis muscle. The tibial division exited inferior to the piriformis, while the common fibular division passed through the muscle belly, effectively splitting the piriformis into superior and inferior bellies. In addition, a neurovascular bundle, including a branch of the inferior gluteal nerve and accompanying veins, was observed emerging from the region of the muscle split. These findings are illustrated in Figure 1 and Figure 2. 

Further dissection revealed a vascular anomaly involving the gluteal arteries. The superior gluteal nerve and artery were located in their expected position above the piriformis muscle. However, a superficial branch of the superior gluteal artery was observed curving around the piriformis and forming an anastomosis with the inferior gluteal artery, as shown in Figure 2.

**Figure 3:**
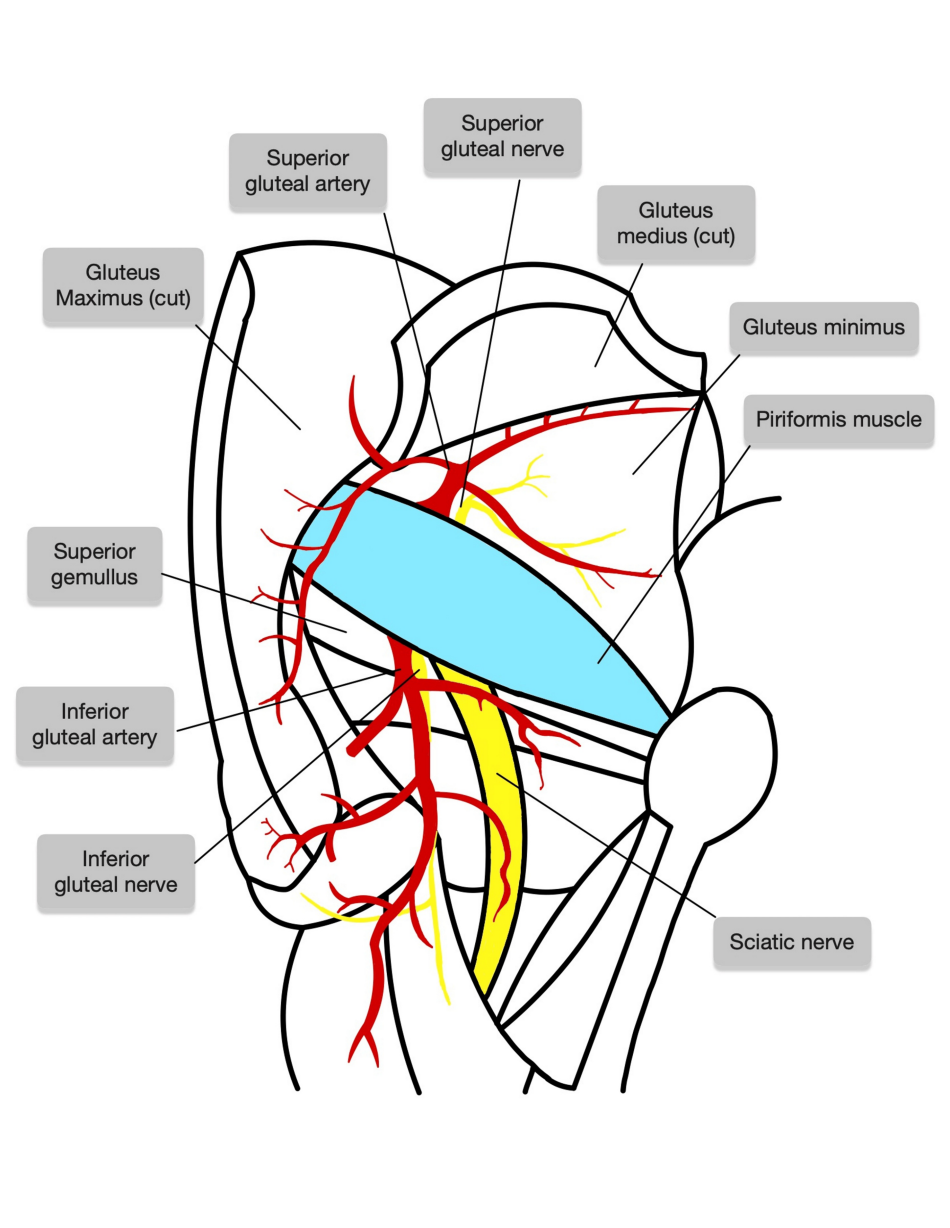
Schematic of the typical anatomical pattern of the piriformis muscle, and superior and inferior gluteal nerves and arteries. This schematic shows the superior gluteal nerve and artery, and the inferior gluteal nerve and artery, which exit superiorly and inferiorly to a single belly of the piriformis muscle, respectively. The sciatic nerve also exits inferior to the piriformis muscle. Figure created by Joshua L. Miles using Goodnotes™ (Goodnotes Limited, London, UK)

The right gluteal region exhibited typical anatomy without notable variation. The schematic of the typical gluteal pattern is shown in Figure 3. The combination of these concurrent neural and vascular anomalies in a single gluteal region makes this cadaveric case particularly rare.

## Discussion

The sciatic nerve, formed from L4-S3, exits the pelvis below the piriformis and travels down the posterior thigh before dividing into the tibial and common fibular nerves near the popliteal fossa [1]. However, numerous deviations from this classic pathway have been documented [4]. The nerve may divide proximally, either within or above the pelvis, or more distally within the thigh [4]. In some cases, one or both branches deviate from the typical path and instead pierce or pass around the piriformis muscle, creating potential sites of entrapment [4].

Beaton and Anson’s six-type classification remains the most widely cited framework for describing sciatic nerve-piriformis anatomical variations [8]. The most common pattern, Type I, occurs in about 87% of individuals and features an undivided sciatic nerve passing anterior and inferior to the piriformis muscle [8]. In Type II, present in roughly 13% of cases, the common fibular division pierces the piriformis while the tibial division follows its normal course beneath the muscle [8]. The anatomical configuration observed in our cadaver aligns with this Type II variant.

In addition to the sciatic nerve anomaly, our dissection revealed a variation in the inferior gluteal nerve. One study reported that only 17 out of 112 cadaveric specimens demonstrated the inferior gluteal nerve passing through the piriformis muscle. In all of those cases, the common fibular nerve accompanied it [5]. This may suggest a shared developmental origin or a structural relationship between the two nerves in variant anatomy. Another case report described a similar dual variation, in which both the inferior gluteal nerve and common fibular nerve coursed between the superior and inferior bellies of a split piriformis in 3.33% of gluteal regions examined [9]. Though infrequent, such variants can carry significant clinical implications. 

Entrapment of either the common fibular division of the sciatic nerve or the inferior gluteal nerve as they traverse the piriformis can contribute to extraspinal causes of sciatica or deep gluteal syndrome, both characterized by buttock pain and radiating neuropathic symptoms [9]. In a recent study, researchers used MR neurography to demonstrate that sciatic nerve variants, particularly those with aberrant trajectories through or around the piriformis, may contribute to cases of sciatica resistant to conventional spine-focused interventions [8]. This highlights the importance of considering peripheral nerve entrapment in the differential diagnosis of treatment-resistant or undiagnosed neuropathies. 

To fully understand why such variations occur, it is important to consider the embryologic origins of both the piriformis muscle and the sciatic nerve. The development of the piriformis muscle and sciatic nerve reflects the coordinated evolution of musculoskeletal and neural elements within the lower limb bud. The lower limb begins forming during the fourth week of embryogenesis as a protrusion of mesenchyme derived from the lateral plate mesoderm, covered by ectoderm and invaded by myogenic precursor cells originating from the somites. These myogenic cells migrate into the limb bud and gluteal mesenchyme, where they form primitive muscle masses that later differentiate into distinct muscles of the thigh and gluteal region [3,10].

By approximately 11-12 weeks of gestation, the gluteal muscles become individually identifiable, including the piriformis, which arises from the anterior surface of the sacrum and inserts into the greater trochanter of the femur. It is derived from the dorsal muscle mass and represents the deep external rotators of the hip. The final positioning of the piriformis as it passes through the greater sciatic foramen reflects the complex rotation and rearrangement of the lower limb during early fetal development [10].

During this same developmental window, the sciatic nerve, the largest peripheral nerve in the human body, forms from the lumbosacral plexus, which arises from the ventral rami of spinal nerves L4-S3. Its axons extend distally from the neural tube, guided by molecular cues, to innervate the developing posterior thigh and lower leg muscles. The sensory ganglia that accompany these fibers are derived from neural crest cells that migrate along the forming vertebral axis and contribute to spinal ganglia and peripheral nerve structures [11].

A human fetal dissection study by Sulak et al. (2014) provided detailed morphometric data showing that the sciatic nerve exits the pelvis below the piriformis in approximately 98% of fetuses. The authors concluded that both the relative size and topographic relationship of the sciatic nerve and piriformis are established early in the second trimester, suggesting that later variations (e.g., the nerve piercing or splitting around the piriformis) result from slight deviations in the migration or branching of the lumbosacral nerve fibers during development [12]

Further developmental studies in animal models have demonstrated that myelination and vascularization of the sciatic nerve occur progressively, beginning near embryonic day 16 in mice and continuing postnatally, indicating that the functional maturation of the nerve parallels its structural refinement [13].

Thus, the piriformis and the sciatic nerve share an intimate embryologic origin and a common spatial evolution. The muscle’s formation from the dorsal muscle mass and the nerve’s pathfinding from the lumbosacral plexus together define their close anatomical relationship in the adult. Variations such as the sciatic nerve piercing or dividing around the piriformis are likely minor developmental shifts during the differentiation and migration stages of limb and plexus formation [12].

Beyond neural variations, a concurrent vascular anomaly was identified, highlighting the intertwined development of neurovascular structures in the gluteal region. A superficial branch of the superior gluteal artery was observed curving around the piriformis muscle and forming an anastomosis with the inferior gluteal artery. Though rare, similar vascular variations have been reported [1]. One case described an atypical course of the inferior gluteal artery, which pierces the superior portion of the piriformis muscle, suggesting that altered spatial relationships may force the artery through the muscle, thereby predisposing it to compression or entrapment [14]. The clinical consequences of such variations may be significant. The inferior gluteal artery contributes to the vascular supply of the femoral head via anastomoses with the medial femoral circumflex artery. Compression or distortion of this vessel could theoretically impair retrograde flow and contribute to idiopathic hip pain in patients without spinal or intra-articular pathology [15].

From a surgical standpoint, the implications of these findings are equally important. In posterior approaches to the hip, one study demonstrated that incisions made more than 5 cm from the tip of the greater trochanter increase the risk of damaging the inferior gluteal nerve, particularly at the site where it innervates the gluteus maximus [16]. In cases involving atypical nerve courses, as observed in our dissection, the risk of iatrogenic nerve injury may be even greater. As such, modifications to standard incision techniques should be considered to avoid zones of high anatomical variability. Moreover, vascular anomalies must be considered during procedures such as buttock flap harvests, total hip arthroplasty, or sciatic nerve decompression, as unanticipated vascular patterns may complicate hemostasis or increase the risk of arterial injury.

Our literature search did not identify any prior documentation of a case in which the common fibular division of the sciatic nerve, a branch of the inferior gluteal nerve, and an anastomotic connection between the superior and inferior gluteal arteries all coursed through, or in close relation to, the piriformis muscle. This case highlights the importance of recognizing complex anatomical variability in the deep gluteal region. In selected cases, preoperative imaging, such as MR neurography or angiography, may help identify such anomalies and minimize intraoperative risk. A comprehensive understanding of these variations is crucial for accurate diagnosis, safe surgical practice, and effective anatomical education.

## Conclusions

This case report highlights a rare anatomical presentation involving concurrent variations of the sciatic and inferior gluteal nerves, as well as an unusual anastomosis between branches of the superior and inferior gluteal arteries. While individual nerve variations in the gluteal region are relatively common, their simultaneous occurrence, along with an associated vascular anomaly, is seldom reported. These findings underscore the importance of awareness and identification of anatomical variants during surgical procedures, nerve blocks, and radiological assessments. Detailed anatomical knowledge of such variations can help reduce the risk of iatrogenic injury and improve clinical outcomes. This case also reinforces the educational value of cadaveric dissection in identifying and documenting anatomical diversity relevant to both clinical practice and research.

## References

[1] Giuffre BA, Black AC, Jeanmonod R (2025 Jan-). Anatomy, sciatic nerve. StatPearls [Internet].

[2] Merryman J, Asuka E, Black AC (2025 Jan-). Anatomy, abdomen and pelvis: inferior gluteal nerve. StatPearls [Internet].

[3] Chang C, Jeno SH, Varacallo MA (2025 Jan-). Anatomy, bony pelvis and lower limb: piriformis muscle. StatPearls [Internet].

[4] Eastlack J, Tenorio L, Wadhwa V, Scott K, Starr A, Chhabra A (2017). Sciatic neuromuscular variants on MR neurography: frequency study and interobserver performance. Br J Radiol.

[5] Tillmann B (1979). [Variations in the pathway of the inferior gluteal nerve (author's transl)]. Anat Anz.

[6] Al Talalwah W (2013). The Vascular Variability of The Iliac System and Clinical Diagnosis in Radiology and Neurology [DOCTORAL THESIS]‏. University of Dundee.

[7] Al-Talalwah W (2015). The vascular supply of hip joint and its clinical significant. Int J Morphol.

[8] Bharadwaj UU, Varenika V, Carson W (2023). Variant sciatic nerve anatomy in relation to the piriformis muscle on magnetic resonance neurography: a potential etiology for extraspinal sciatica. Tomography.

[9] Kasapuram D, Ganapathy A, Harisha K, Bhukya S, Rani N, Singh S (2021). Neuromuscular variations in the gluteal region - embryological basis and clinical significance. Clin Ter.

[10] Moore KL, Persaud TVN, Torchia MG (2016). The Developing Human: Clinically Oriented Embryology (10th Ed.). https://www.google.co.in/books/edition/_/lC7QoQEACAAJ?hl=en&sa=X&ved=2ahUKEwidnuzC-Z6RAxXVT2wGHZyQG8oQre8FegQIDRAL.

[11] Sadler TW (2020). Langman’s Medical Embryology. https://openlibrary.org/books/OL25263957M/Langman%27s_medical_embryology.

[12] Sulak O, Sakalli B, Ozguner G, Kastamoni Y (2014). Anatomical relation between sciatic nerve and piriformis muscle and its bifurcation level during fetal period in human. Surg Radiol Anat.

[13] Chen Y, Shang T, Sun J (2024). Characterization of sciatic nerve myelin sheath during development in C57BL/6 mice. Eur J Neurosci.

[14] Anetai H, Tokita K, Kojima R, Toriumi T, Kageyama I, Kumaki K (2024). An atypical inferior gluteal artery passing through the piriformis muscle. Surg Radiol Anat.

[15] Grose AW, Gardner MJ, Sussmann PS, Helfet DL, Lorich DG (2008). The surgical anatomy of the blood supply to the femoral head: description of the anastomosis between the medial femoral circumflex and inferior gluteal arteries at the hip. J Bone Joint Surg Br.

[16] Ling ZX, Kumar VP (2006). The course of the inferior gluteal nerve in the posterior approach to the hip. J Bone Joint Surg Br.

